# Association between inter-leg blood pressure difference and cardiovascular outcome in patients undergoing percutaneous coronary intervention

**DOI:** 10.1371/journal.pone.0257443

**Published:** 2021-10-15

**Authors:** Inki Moon, Hack-Lyoung Kim, Woo-Hyun Lim, Jae-Bin Seo, Joo-Hee Zo, Myung-A Kim, Sang-Hyun Kim

**Affiliations:** 1 Division of Cardiology, Department of Internal Medicine, Soonchunhyang University Bucheon Hospital, Bucheon, Republic of Korea; 2 Division of Cardiology, Department of Internal Medicine, Boramae Medical Center, Seoul National University College of Medicine, Seoul, Republic of Korea; Shanghai Institute of Hypertension, CHINA

## Abstract

**Background:**

Although the inter-arm blood pressure (BP) difference has been advocated to be associated with cardiovascular events, the implication of inter-leg BP difference has not been well established. This study was conducted to investigate whether inter-arm and -leg BP differences have prognostic value in patients undergoing percutaneous coronary intervention (PCI).

**Methods:**

In this prospective study, we consecutively enrolled 667 patients who underwent PCI. Both arm and leg BPs were measured at the day after PCI. The primary outcome was a major adverse cardiovascular event (MACE) including cardiac death, acute coronary syndrome, coronary revascularization, stroke, and hospitalization for heart failure during the follow-up period.

**Results:**

Mean age was 64.0±11.1 years old, and males were predominant (70.5%). During a mean follow-up period of 3.0 years, MACE occurred in 209 (31.3%) patients. The inter-leg systolic BP difference (ILSBPD) was significantly higher in patients with MACE than those without (9.9±12.3 vs. 7.2±7.5 mmHg, *P* = 0.004). The inter-arm systolic BP difference was not significantly different between patients with and without MACE (*P* = 0.403). In multivariable Cox regression analysis, increased ILSBPD was independently associated with the development of MACE (per 5 mmHg; hazard ratio, 1.07; 95% confidence interval, 1.01–1.14). The inter-arm systolic BP difference was not associated with MACE in the multivariable analysis.

**Conclusion:**

Increased ILSBPD was independently associated with worse cardiovascular outcomes after PCI. As ILSBPD is easy to measure, it may be helpful in the risk stratification of patients undergoing PCI.

## Introduction

The burden of cardiovascular (CV) disease continues to rise, and it has become the leading cause of death worldwide [1]. Therefore, early identification of patients with high risk for CV disease and more aggressive management for these patients are essential. For assessing CV risk, clinicians have used data from blood pressure (BP), body mass index, laboratory and imaging tests, and risk scoring systems such as the Framingham risk score [2,3]. Despite many efforts using these various methods to find high-risk individuals, there has been limitations in the risk stratification and prevention of CV events [3,4]. Thus, there is a need to discover a new risk indicator.

The cost-effectiveness differs among examinations according to patient situation, but there is an argument that BP measurement is the most inexpensive and effective way to estimate individuals’ risk [3]. After recognition of the prevalence and clinical implication of inter-arm systolic BP difference (IASBPD) [5,6], there has been growing evidence for the prognostic capability of IASBPD. Greater IASBPD is closely linked to coronary and peripheral artery diseases (PAD) [7,8], CV events [9,10], and mortality [11]. Furthermore, based on various previous studies, there were some suggestions that a cutoff value of IASBPD has a significant clinical impact [11]. On the other hand, data on the clinical implication of inter-leg BP difference has been limited. Only a few studies have reported that inter-leg systolic BP difference (ILSBPD) is associated with PAD [12], stroke [13], and mortality [14,15].

The prevalence of coronary artery disease has increased, and percutaneous coronary intervention (PCI) is one of the important treatment strategies for coronary artery disease. In Korea, about 50,000 patients underwent PCI every year [16]. As such patients are at high risk of CV events, we should pay more attention to these patients and make efforts to prevent recurrence. Therefore, this study was performed to investigate whether inter-arm and -leg BP differences have prognostic value in patients undergoing PCI.

## Materials and methods

This single-center, prospective study was performed at a general hospital located in a large city (Seoul, Korea). From April 2012 to August 2015, we consecutively recruited patients undergoing PCI with a drug-eluting stent. All the study patients underwent bilateral arm and leg BP measurement during stabilized condition at the day after PCI. From the total of initially screened 717 patients, those who had the following conditions were excluded: 1) failed PCI, 2) previous percutaneous transluminal angioplasty for peripheral artery disease, 3) ankle-brachial index over 1.4 or less than 0.9, and 4) malignancy. After these exclusions, this cohort was made up a total of 667 patients. The study protocol was approved by the Institutional Review Board of Boramae Medical Center (Seoul, Korea), and written informed consent was obtained from each study subject.

We obtained the patients’ age and sex information. Body mass index was calculated by dividing body weight by squared height (kg/m^2^). Data on underlying medical conditions, including hypertension, diabetes mellitus, hyperlipidemia, coronary artery disease, atrial fibrillation, chronic kidney disease, previous history of stroke, and smoking status, were acquired. We defined the hypertension as systolic blood pressure ≥140 mmHg, diastolic blood pressure ≥90 mmHg, or current medications. Diabetes mellitus were defined as fasting blood glucose ≥126 mg/dL, glycated hemoglobin ≥6.5%, or use of anti-diabetic medications. Coronary artery disease included a history of myocardial infarction or coronary revascularization. Atrial fibrillation was defined as a history of any atrial fibrillation which was documented by electrocardiography. Patients with chronic kidney disease were those whose estimated glomerular filtration rate (eGFR) was below 60 mL/min/1.73 m^2^. We obtained the history of the previous stroke from the medical records of the patients. A patient who smoked within 12 months was defined as a current smoker. After 12 hours of fasting, blood was sampled for the determination of hemoglobin, estimated glomerular filtration rate, total cholesterol, low-density lipoprotein cholesterol, high-density lipoprotein cholesterol, and triglyceride. Left ventricular ejection fraction was calculated using Simpson’s biplane method in transthoracic echocardiography.

The day after PCI, 4-extremity BP were measured simultaneously. After 5 minutes of rest, the test was performed in the supine position. BP was measured with a noninvasive vascular device (VP-1000; Colin Co. Ltd., Komaki, Japan) [14,17]. With cuffs on both arms and ankles, BP were measured by the oscillometric method. Trained technicians and physicians measured the BPs according to the manufacturer’s recommendations. The device automatically measures the BPs twice simultaneously, deletes the first measurement, and then stores only the second value in the database. If first and second BP measurements differ by more than 15 mmHg, the inspector made third measurements. The inter-arm and -leg BP differences were calculated by absolute differences between the right and left arm BPs and between the right and left ankle BPs. Both were separately calculated in systolic and diastolic BPs.

The study outcome was a major adverse cardiovascular event (MACE). The MACE was a composite of cardiovascular death, nonfatal acute coronary syndrome, coronary revascularization including PCI and coronary artery bypass graft surgery, non-fatal ischemic stroke, and hospitalization for cardiovascular causes. Cardiovascular death included the death caused by acute coronary syndrome, ventricular arrhythmia, heart failure, or unexplained sudden death. Nonfatal acute coronary syndrome was identified by a cardiologists using conclusive evidence, which was provided from an elevation of cardiac enzymes or ST-segment changes. A diagnosis of ischemic stroke was confirmed by a neurologist based on physical examination and brain imaging modalities. Hospitalization for the cardiovascular causes included unplanned admission for heart failure, coronary artery disease, atrial fibrillation, other arrhythmias, or transient ischemic attack. We regularly followed up the study subjects for the occurrence of MACE using hospital records; telephone interviews were used for the follow-up, if necessary.

Continuous variables are presented as mean ± standard deviation (SD), and categorical variables as percentages. Student’s *t* test was used to compare continuous variables, and the Chi-square test or Fisher’s exact test was used to compare categorical variables between 2 groups. Using the Cox-proportional regression hazard method, we identified predictors for MACE among clinical and laboratory factors as well as among inter-arm and inter-leg BP differences. The confounders, which had *P* values of <0.1 in univariable analysis, were adjusted to investigate the association of inter-leg BP difference and MACE. The confounding factors were age, sex, hypertension, diabetes mellitus, previous coronary artery disease, atrial fibrillation, chronic kidney disease, previous stroke, current smoking, and hemoglobin. The optimal cutoff value of inter-leg systolic BP difference (ILSBPD) for MACE was estimated using maximally selected log-rank statistics [18]. After revealing the optimal cutoff value of ILSBPD, a Kaplan-Meier survival curve with the log-rank test was used to compare the occurrence of MACE between the 2 groups stratified according to ILSBPD. To investigate the clinical difference of the 2 groups divided according to the ILSBPD cutoff value, the multivariable Cox-proportional regression hazard method was repeated with the same risk factors. We also evaluated the prognostic value of ILSBPD when added to clinical risk factors predicting MACE by using global Chi-square scores. A *p* value of <0.05 was used to verify statistical significance. All statistical analyses were conducted using R version 3.4.3 (http://www.r-projec-t.org).

## Results

Among a total of 667 study patients, 209 (31.3%) had MACE, including cardiovascular death (n = 3, 0.4%), non-fatal acute coronary syndrome (n = 28, 4.2%), coronary revascularization (n = 52, 7.8%), non-fatal stroke (n = 28, 4.2%), and hospitalization for cardiovascular causes (n = 98, 14.7%) during a mean follow-up period of 3.0 years (median 3.26 years; interquartile range 1.55–4.27 years). **Table 1** shows the baseline characteristics of the patients and differences between groups with and without MACE. Patients with MACE were older (67.3±10.5 vs. 62.5±11.0 years, *P*<0.001) and had a higher prevalence of diabetes mellitus, coronary artery disease, atrial fibrillation, chronic kidney disease, history of the previous stroke, and the multi-vessel disease than those without. In laboratory findings, patients with MACE had lower levels of hemoglobin and eGFR than those without. The detailed information of MACE was presented in **S1 Table**.

**Table 1:** Baseline characteristics according to major adverse cardiovascular events.

	Total (n = 667)	MACE (+) (n = 209)	MACE (-) (n = 458)	*P* value
**Clinical factors**				
Age (years)	64.0 ± 11.1	67.3 ± 10.5	62.5 ± 11.0	<0.001
Male sex	470 (70.5)	138 (66.0)	332 (72.5)	0.108
Body mass index (kg/m^2^)	24.7 ± 3.5	24.5 ± 3.5	24.8 ± 3.5	0.327
Hypertension	416 (62.4)	141 (67.5)	275 (60.0)	0.080
Diabetes Mellitus	205 (30.7)	88 (42.1)	117 (25.5)	<0.001
Previous coronary artery disease	122 (18.3)	51 (24.4)	71 (15.5)	0.008
Atrial fibrillation	43 (6.4)	20 (9.6)	23 (5.0)	0.041
Chronic kidney disease	36 (5.4)	22 (10.5)	14 (3.1)	<0.001
Previous stroke	52 (7.8)	27 (12.9)	25 (5.5)	0.001
Current smoker	202 (30.3)	53 (25.4)	149 (32.6)	0.072
Diagnosis for PCI				0.049
Silent ischemia	24 (3.6)	13 (6.2)	11 (2.4)	
Stable angina	98 (14.7)	30 (14.4)	68 (14.8)	
Unstable angina	314 (47.1)	97 (45.5)	217 (47.4)	
NSTEMI	112 (16.8)	40 (19.1)	72 (15.7)	
STEMI	119 (17.8)	29 (13.9)	90 (19.7)	
Coronary artery underwent PCI				
Left main	62 (9.3)	23 (11.0)	39 (8.5)	0.377
Left anterior descending	438 (65.7)	137 (65.6)	301 (65.7)	0.999
Left circumflex	216 (32.4)	68 (32.5)	148 (32.3)	0.999
Right coronary artery	244 (36.6)	85 (40.7)	159 (34.7)	0.163
Multi-vessel disease	473 (70.9)	166 (79.4)	307 (67.0)	0.001
**Laboratory findings**				
Hemoglobin (g/dL)	13.3 ± 2.3	12.7 ± 2.3	13.5 ± 2.2	<0.001
Estimated GFR (mL/min)	81.4 ± 25.3	74.3 ± 30.1	84.7 ± 22.0	<0.001
Total cholesterol (mg/dL)	167.3 ± 48.0	162.7 ± 47.4	169.3 ± 48.1	0.124
LDL cholesterol (mg/dL)	99.6 ± 37.5	96.7 ± 39.9	101.0 ± 36.3	0.194
HDL cholesterol (mg/dL)	42.1 ± 12.0	41.2 ± 11.8	42.4 ± 12.2	0.239
Triglyceride (mg/dL)	126.4 ± 78.4	124.6 ± 80.0	127.2 ± 77.8	0.699
LVEF (%)	61.5 ± 11.1	60.7 ± 11.5	61.8 ± 11.0	0.264
**Medication**				
Aspirin	659 (98.9)	206 (98.6)	453 (99.1)	0.804
Clopidogrel	635 (95.3)	201 (96.2)	434 (95.0)	0.626
Beta-blocker	514 (77.2)	169 (80.9)	345 (75.5)	0.152
RAS blocker	508 (76.3)	159 (76.1)	349 (76.4)	0.999
Calcium channel blocker	164 (24.6)	59 (28.2)	105 (23.0)	0.173
Statin	636 (95.5)	196 (93.8)	440 (96.3)	0.214

Data is shown by mean ± SD or number (%). GFR, glomerular filtration rate; HDL, high-density lipoprotein; LDL, low-density lipoprotein; NSTEMI, non-ST elevation myocardial infarction; PCI, percutaneous coronary intervention; RAS, renin-angiotensin system; STEMI, ST elevation myocardial infarction; VD, vessel disease.

In 4-extremity BP measurements (**Table 2**), right-arm systolic BP was higher in patients with MACE (123.1±18.1 vs. 119.2±15.6 mmHg, *P* = 0.008). The IASBPD and the inter-arm diastolic BP difference (IADBPD) were not different between the 2 groups. The ILSBPD (9.9±12.3 vs. 7.2±7.5 mmHg, *P* = 0.004) and inter-leg diastolic BP difference (ILDBPD) (4.6±4.9 vs. 3.6±4.9 mmHg, *P* = 0.015) were significantly higher in patients with MACE than those without. There were 88 patients (13.2%) whose ILSBPD was greater than 15 mmHg and 25 patients (3.7%) whose ILDBPD was greater than 15 mmHg.

**Table 2:** Four-extremity blood pressure measurements according to major adverse cardiovascular events.

	Total (n = 667)	MACE (+) (n = 209)	MACE (-) (n = 458)	*P* value
Right arm systolic BP (mmHg)	120.4 ± 16.5	123.1 ± 18.1	119.2 ± 15.6	0.008
Right arm diastolic BP (mmHg)	71.8 ± 10.1	71.3 ± 10.6	72.0 ± 9.8	0.401
Left arm systolic BP (mmHg)	119.8 ± 16.4	121.5 ± 17.8	119.1 ± 15.7	0.179
Left arm diastolic BP (mmHg)	71.9 ± 9.9	71.3 ± 10.4	72.1 ± 9.7	0.378
Right leg systolic BP (mmHg)	138.5 ± 24.8	139.4 ± 29.7	138.2 ± 22.3	0.589
Right leg diastolic BP (mmHg)	71.5 ± 11.6	70.2 ± 12.7	72.0 ± 11.0	0.079
Left leg systolic BP (mmHg)	137.8 ± 25.3	138.6 ± 29.3	137.4 ± 23.3	0.590
Left leg diastolic BP (mmHg)	71.9 ± 12.1	71.3 ± 12.5	72.1 ± 11.9	0.401
Inter-arm SBP difference (mmHg)	3.9 ± 4.2	4.1 ± 5.2	3.8 ± 3.7	0.403
Inter-arm DBP difference (mmHg)	2.9 ± 2.4	2.8 ± 2.9	2.9 ± 2.2	0.722
Inter-leg SBP difference (mmHg)	8.0 ± 9.4	9.9 ± 12.3	7.2 ± 7.5	0.004
Inter-leg DBP difference (mmHg)	3.9 ± 4.9	4.6 ± 4.9	3.6 ± 4.9	0.015

Data is shown by mean ± SD. BP, blood pressure; DBP, diastolic blood pressure; SBP, systolic blood pressure.

To identify predictors for the occurrence of MACE, we analyzed the prognostic value of baseline clinical characteristics, and inter-arm and -leg BP differences using the Cox proportional hazard regression method (**Table 3**). Age, diabetes mellitus, coronary artery disease, atrial fibrillation, chronic kidney disease, previous stroke, and hemoglobin level showed associations with the MACE. In extremities BP differences, both ILSBPD and ILDBPD showed a significant association, while inter-arm BP difference had no significant correlation with MACE. After adjustment for potential confounders, ILSBPD still had a significant association with MACE (per 5 mmHg increase; hazard ratios [HR], 1.07; 95% confidence interval [CI], 1.01–1.14; *p* = 0.028), but ILDBPD did not (*p* = 0.805). These results were consistent after adjusting IASBPD or IADBPD (**S2 Table).** And, after additional analyzing with high right-arm systolic BP (≥140 mmHg) and high right-arm diastolic BP (≥90 mmHg), the ILSBPD still significantly associated with MACE (HR 1.07; 95% CI 1.00–1.13; P = 0.036 with high systolic BP, and HR 1.07; 95% CI 1.01–1.13; P = 0.031 with high diastolic BP).

**Table 3:** Predictors for major adverse cardiovascular events.

	Univariable analysis			Multivariable adjusted analysis					
	HR	95% CI	*P*	HR*	95% CI	*P*	HR^†^	95% CI	*P*
**Risk factors**									
Age (per years)	1.04	1.03–1.06	<0.001	1.03	1.01–1.04	<0.001	1.03	1.02–1.05	<0.001
Male sex	0.77	0.58–1.03	0.076	0.92	0.67–1.28	0.620	0.95	0.68–1.33	0.746
Hypertension	1.28	0.96–1.72	0.091	0.89	0.65–1.22	0.482	0.93	0.68–1.27	0.626
Diabetes mellitus	1.77	1.35–2.33	<0.001	1.53	1.15–2.04	0.004	1.57	1.18–2.09	0.002
Previous coronary artery disease	1.57	1.14–2.15	0.005	1.29	0.93–1.79	0.130	1.26	0.91–1.76	0.161
Atrial fibrillation	2.06	1.30–3.27	0.002	1.67	1.04–2.69	0.033	1.69	1.05–2.72	0.032
Chronic kidney disease	2.31	1.49–3.60	<0.001	1.47	0.90–2.39	0.128	1.57	0.97–2.54	0.069
Previous stroke	1.96	1.31–2.94	0.001	1.70	1.13–2.57	0.012	1.69	1.12–2.55	0.013
Current smoker	0.73	0.54–1.00	0.050	1.07	0.76–1.50	0.700	1.08	0.77–1.51	0.670
Hemoglobin	0.89	0.85–0.94	<0.001	0.95	0.90–1.02	0.150	0.95	0.89–1.01	0.122
**Inter-arm and -leg BP difference **									
Arm SBP Difference, per 5 mmHg	1.09	0.93–1.28	0.275						
Arm DBP Difference, per 5 mmHg	0.95	0.70–1.29	0.746						
Leg SBP Difference, per 5 mmHg	1.13	1.06–1.19	<0.001	1.07	1.01–1.14	0.028			
Leg DBP Difference, per 5 mmHg	1.10	1.00–1.20	0.048				1.01	0.91–1.13	0.805

BP, blood pressure; CI, confidence interval; DBP, diastolic blood pressure; HR, hazard ratio; SBP, systolic blood pressure.

^*^Adjusted with traditional risk factors, and leg SBP difference.

^**†**^Adjusted with traditional risk factors, and leg DBP difference.

**Fig 1:**
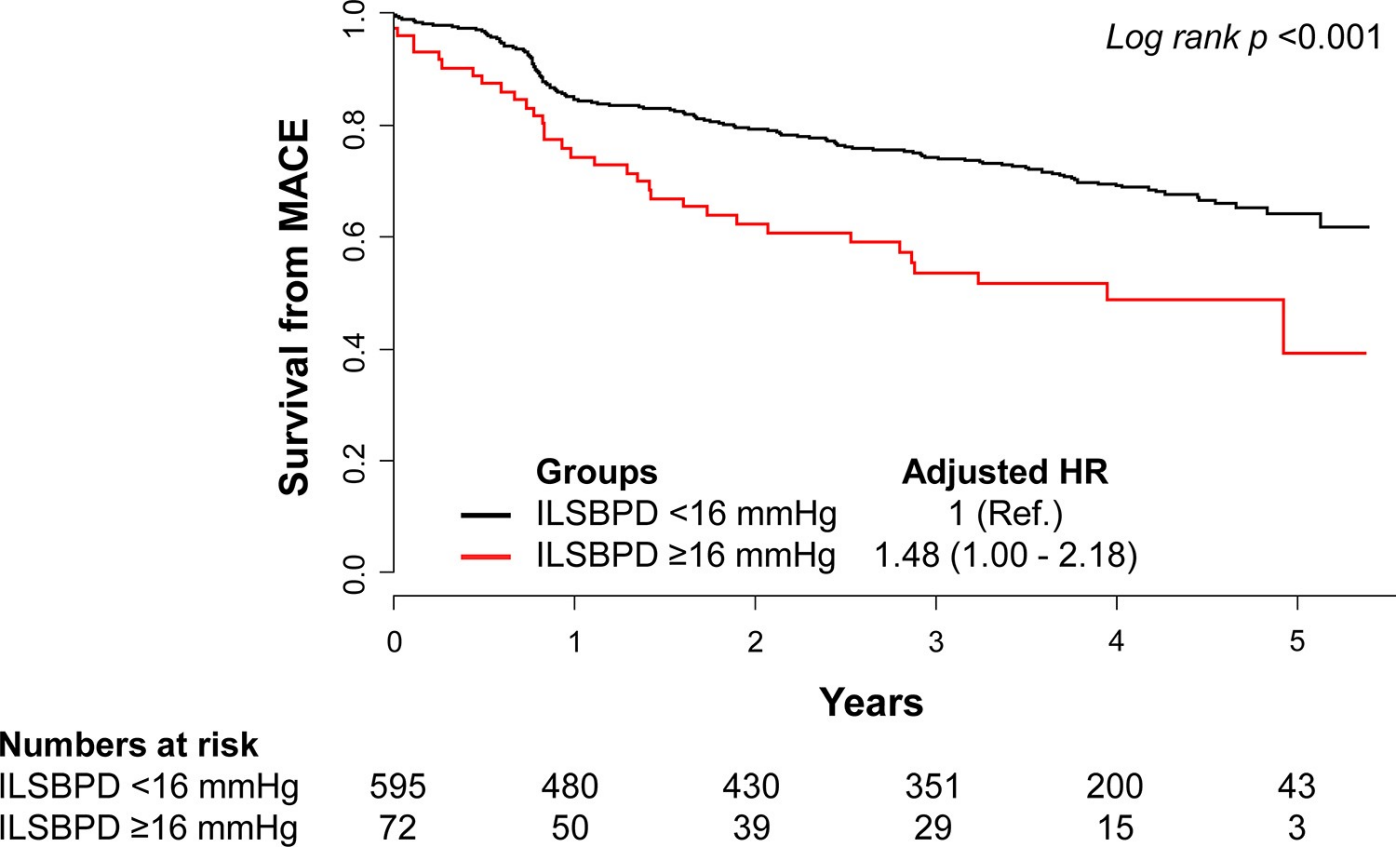
Kaplan-Meier curves for MACE and risk of MACE according to ILSBPD. The curves for survival from MACE are presented according to ILSBPD (<16 vs. ≥16 mmHg). Abbreviation: HR, hazard ratios; ILSBPD, inter-leg systolic blood pressure difference; MACE, major adverse cardiovascular events.

The optimal cutoff value of ILSBPD that maximized the log-rank statistic was 16 mmHg to predict the occurrence of MACE (*P* = 0.01) (**S1 Fig**). After dividing the patients into 2 groups according to the cutoff ILSBPD value of 16 mmHg, the higher ILSBPD (≥16 mmHg) group was older and had lower body mass indices, and higher prevalence rates of hypertension, diabetes mellitus and chronic kidney disease than in the lower ILSBPD (<16 mmHg) group (**S3 Table**). Also, the higher ILSBPD group showed lower hemoglobin and low-density lipoprotein cholesterol levels. The higher ILSBPD group showed significantly higher incidences of MACE than the lower ILSBPD group (19.4 vs. 9.7 per 100 person-year, *P*<0.001, **S2 Fig**). In multivariable Cox proportional hazard regression analysis, the higher ILSBPD group showed a significantly higher risk of MACE than the lower ILSBPD group (HR, 1.48; 95% CI, 1.00–2.18; *P* = 0.048) (**Table 4**). Kaplan-Meier survival curves for MACE according to ILSBPD are presented in **Fig 1**.

**Table 4:** Cox-proportional hazard ratios for major adverse cardiovascular events in higher ILSBPD group.

	Univariable analysis			Multivariable adjusted analysis		
	HR	95% CI	*P*	HR	95% CI	*P*
**Risk factors**						
Age (per years)	1.04	1.03–1.06	<0.001	1.03	1.01–1.04	<0.001
Male sex	0.77	0.58–1.03	0.076	0.93	0.67–1.29	0.664
Hypertension	1.28	0.96–1.72	0.091	0.90	0.66–1.24	0.529
Diabetes mellitus	1.77	1.35–2.33	<0.001	1.56	1.17–2.07	0.002
Coronary artery disease	1.57	1.14–2.15	0.005	1.25	0.90–1.74	0.180
Atrial fibrillation	2.06	1.30–3.27	0.002	1.72	1.07–2.77	0.025
Chronic kidney disease	2.31	1.49–3.60	<0.001	1.46	0.89–2.39	0.133
Previous stroke	1.96	1.31–2.94	0.001	1.69	1.12–2.55	0.013
Current smoker	0.73	0.54–1.00	0.050	1.09	0.77–1.02	0.633
Hemoglobin	0.89	0.85–0.94	<0.001	0.95	0.89–1.02	0.142
High ILSBPD (≥ 16 mmHg)	1.93	1.33–2.78	<0.001	1.48	1.00–2.18	0.048

CI, confidence interval; HR, hazard ratio; ILSBPD, inter-leg systolic blood pressure difference.

**Fig 2:**
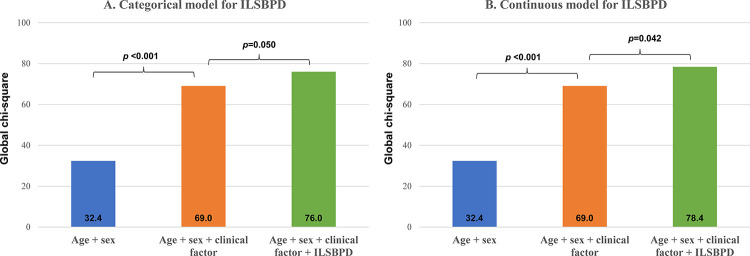
Additional prognostic value of ILSBPD in MACE. The additional prognostic value of ILSBPD is presented in the prediction models with age, sex, and clinical factors. (**A)** the prognostic value of ILSBPD for categorical variables (<16 vs. ≥16 mmHg). (**B)** the prognostic value of ILSBPD for continuous variables. The clinical factors included hypertension, diabetes mellitus, coronary artery disease, atrial fibrillation, chronic kidney disease, previous stroke, current smoker, and hemoglobin. Abbreviation: ILSBPD, inter-leg systolic blood pressure difference; MACE, major adverse cardiovascular events.

**Fig 2** presents the additional prognostic value of ILSBPD in MACE by using global Chi-square scores. For age and sex, global Chi-square scores was 32.4. MACE prediction was significantly improved after adding information on clinical factors (global Chi-square scores, from 32.4 to 69.0; P<0.001). Moreover, the addition of ILSBPD to age, sex, and clinical factors significantly increased prognostic value in MACE for categorical variables (global Chi-square scores, from 69.0 to 76.0; *P* = 0.050) and continuous variables (global Chi-square scores, from 69.0 to 78.4; *P* = 0.042).

## Discussion

This study revealed that ILSBPD was independently associated with MACE in patients who underwent PCI. Patients with ILSBPD ≥16 mmHg had a 1.5-fold higher risk of MACE than those with ILSBPD <16 mmHg ILSBPD even after adjustment for many risk factors. Moreover, adding information on ILSBPD to clinical risk factors significantly increased prediction power for the future development of MACE. To the best of our knowledge, this is the first report to investigate the prognostic value of ILSBPD in patients with coronary artery disease undergoing PCI, suggesting that ILSBPD measurement could be a reliable and convenient method for predicting MACE in high-risk patients.

Many studies have reported that higher IASBPD is associated with PAD, cerebrovascular diseases, cardiovascular diseases, and mortality [5–11]. However, only a few studies have demonstrated the value of ILSBPD in relation to clinical outcomes. Chen *et al*. [15] investigated 210 patients with end-stage renal disease with hemodialysis and found that ILSBPD ≥15 mmHg or ILDBPD ≥10 mmHg is independently associated with increased risk of overall and cardiovascular mortality. Another study of 3,133 elderly Chinese subjects (≥60 years) in the community with 4 years of follow-up found that those with ILSBPD >15 mmHg were at 1.6-fold higher risk of total mortality and at 1.9-fold higher risk of cardiovascular mortality even after controlling for potential confounders [14]. And the ILSBPD is also the independent risk factor for stroke in other Chinese hypertension study [13]. The results of those study are in line with ours. We simultaneously measured and compared both inter-arm and inter-leg BP differences, and first demonstrated that the prognostic value of ILSBPD in patients with documented coronary artery disease.

Another interesting finding of our study is that only ILSBPD had a significant association with MACE, while IASBPD did not. Previous studies showing the evidence of the prognostic value of IASBPD included patients with hypertension [19], outpatients of the cardiology and vascular department [20], or elderly patients [14]. The difference in clinical characteristics of study populations may cause different results. However, there were similar results in the studies using all four-limb BPs. ILSBPD showed a significant association with the prevalence of stroke [13] and cardiovascular mortality [14] in Chinese elderly patients. Additionally, the difference between ILSBPD and IASBPD may be related to the difference in the length of arteries between upper and lower extremities. Longer arteries have more chance of vessel disease such as atherosclerosis, which may affect the burden of vessel disease and the BP difference. Our study investigated patients undergoing PCI, so they may have suffered from more advanced atherosclerosis compared to those of prior studies.

Patients with higher ILSBPD (≥16 mmHg) were older and had higher proportions of hypertension, diabetes mellitus, and chronic kidney disease. The higher risk in patients with higher ILSBPD may improve the prognostic value of ILSBPD in predicting future cardiovascular events. In addition, PAD, a strong prognostic marker of cardiovascular diseases, is usually defined as the vascular disease of the lower extremity arteries; therefore, it is acceptable that ILSBPD has a stronger association with PAD compared to IASBPD [6]. Although we excluded patients with significant PAD (ABI ≤0.9), there was a possibility that they could already have atherosclerotic changes in the lower extremities. Some studies have reported significant prognostic value of ABI even if it is >0.9 [17,21]. Indeed, patients with higher ILSBPD had significantly lower levels of both ABI than those with lower ILSBPD in our study (right ABI: 1.12±0.12 vs. 1.1 ±0.09, P = 0.039; left ABI: 1.10±0.10 vs. 1.15±0.09, P<0.001). Increased ILSBPD may correlate with mild arterial disease in the lower extremities; however, our study cannot fully explain the whole mechanism for the prognostic impact of ILSBPD. Future studies are needed to elucidate mechanisms for the association between ILSBPD and MACE and the different prognostic value of ILSBPD and IASBPD.

The measurement of ILSBPD is simple. Therefore, ILSBPD is very useful as a primary screening test for selecting high-risk patients. If patients undergoing PCI have high ILSBPD, they will need more attention, and intensive treatment and monitoring.

This study has several limitations. First, our study population was limited to patients who underwent PCI and without significant PAD which may make it difficult to generalize our findings to other populations. Secondly, our study design was observational and not fully controlled; therefore, there could be uncontrolled confounding factors even with the effort of adjustment. Third, there could be a concern for including the patients with atrial fibrillation. We repeated the measurements at least three times and used an average of these multiple measurements for advancing the accuracy. Furthermore, an independent association between ILSBPD and MACE still exist in multivariable analysis even after excluding the patients with atrial fibrillation. Finally, we included soft prognostic endpoints to MACE, such as hospitalization for cardiovascular causes, because hard endpoint events were rare in our cohort.

In conclusion, ILSBPD was independently associated with worse cardiovascular outcomes after PCI. As a simple measurement of atherosclerosis, ILSBPD may be useful for the risk stratification of patients undergoing PCI. Further investigations will be needed to validate the prognostic value of ILSBPD in higher risk patients and/or more general population for acceptance in clinics.

## Supporting information

S1 Fig Evaluating the cutoff point of ILSBPD with maximally selected log-rank statistics. The optimal cutoff value of ILSBPD, for predicting the major adverse cardiovascular events, that maximized the log-rank statistic was 16 mmHg (*p* = 0.01).
(DOCX)

S2 Fig The incidence of MACE stratified by ILSBPD. (DOCX)

S1 Table Detailed information of major adverse cardiovascular events. (DOCX)

S2 Table Predictors for major adverse cardiovascular events. (DOCX)

S3 Table Comparison of characteristics between patients with ILSBPD <16mmHg and ILSBPD ≥ 16mmHg. (DOCX)
