# Supplementary material for: Association between inter-leg blood pressure difference and cardiovascular outcome in patients undergoing percutaneous coronary intervention
Source: PLoS One. 2021 Oct 15;16(10):e0257443. doi: 10.1371/journal.pone.0257443 (PMC8519463; doi:10.1371/journal.pone.0257443)
Supplement: S1 Fig — The optimal cutoff value of ILSBPD, for predicting the major adverse cardiovascular events, that maximized the log-rank statistic was 16 mmHg (p = 0.01). (DOCX) [file pone.0257443.s001.docx]

**S1 Figure. Evaluating the cutoff point of ILSBPD with maximally selected log-rank statistics.**

**
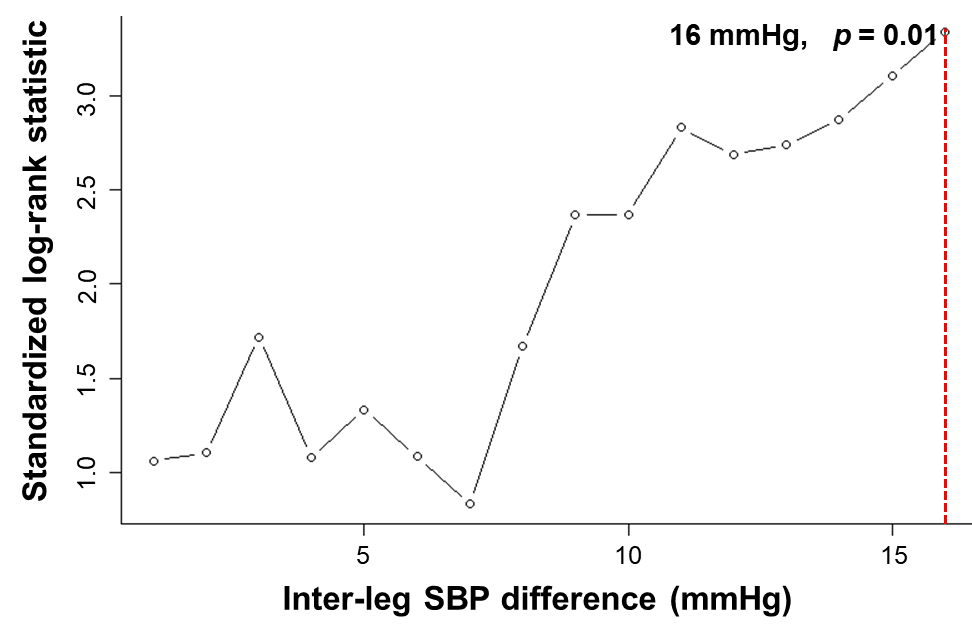
**

The optimal cutoff value of ILSBPD, for predicting the major adverse cardiovascular events, that maximized the log-rank statistic was 16 mmHg (*p* = 0.01).

Abbreviation: ILSBPD, inter-leg systolic blood pressure difference; SBP, systolic blood pressure
