# Supplementary material for: Association between inter-leg blood pressure difference and cardiovascular outcome in patients undergoing percutaneous coronary intervention
Source: PLoS One. 2021 Oct 15;16(10):e0257443. doi: 10.1371/journal.pone.0257443 (PMC8519463; doi:10.1371/journal.pone.0257443)
Supplement: S2 Fig — (DOCX) [file pone.0257443.s002.docx]

**S2 Figure. The incidence of MACE stratified by ILSBPD**

**
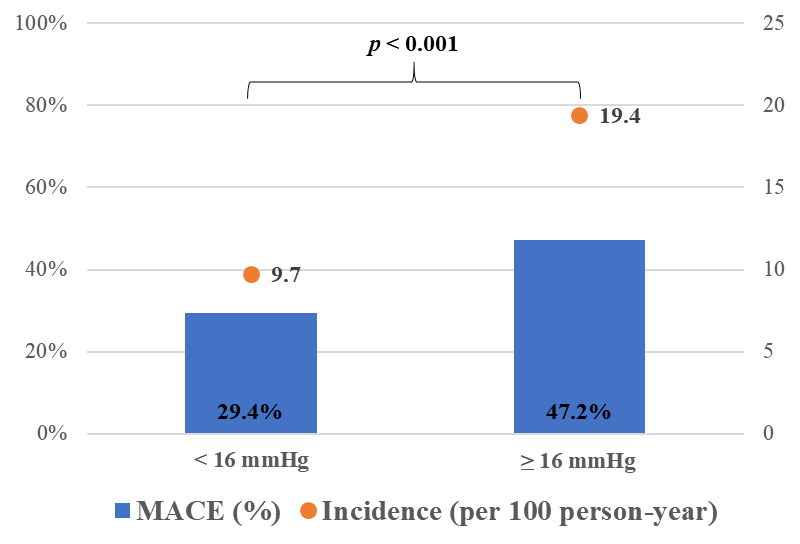
**

Abbreviation: ILSBPD, inter-leg systolic blood pressure difference; MACE, major adverse cardiovascular events
