# Supplementary material for: Association between inter-leg blood pressure difference and cardiovascular outcome in patients undergoing percutaneous coronary intervention
Source: PLoS One. 2021 Oct 15;16(10):e0257443. doi: 10.1371/journal.pone.0257443 (PMC8519463; doi:10.1371/journal.pone.0257443)
Supplement: S1 Table — (DOCX) [file pone.0257443.s003.docx]

**S1 Table. Detailed information of major adverse cardiovascular events**

|  | **MACE (+)** |
| --- | --- |
|  | **N=209** |
| **Cardiovascular death** | 13 (6.2%) |
| **Nonfatal acute coronary syndrome** | 48 (23.0%) |
| **Coronary revascularization** | 90 (43.1%) |
| **Nonfatal ischemic stroke** | 20 (9.6%) |
| **Hospitalization for cardiovascular causes** | 38 (18.2%) |

MACE; major adverse cardiovascular events.
