# Supplementary material for: Association between inter-leg blood pressure difference and cardiovascular outcome in patients undergoing percutaneous coronary intervention
Source: PLoS One. 2021 Oct 15;16(10):e0257443. doi: 10.1371/journal.pone.0257443 (PMC8519463; doi:10.1371/journal.pone.0257443)
Supplement: S2 Table — (DOCX) [file pone.0257443.s004.docx]

**S2 Table. Predictors for major adverse cardiovascular events**

|  | **Univariable analysis** | | |  | **Multivariable adjusted analysis** | | | | | | |
| --- | --- | --- | --- | --- | --- | --- | --- | --- | --- | --- | --- |
|  | **HR** | **95% CI** | ***p*** |  | **HR^*^** | **95% CI** | ***p*** |  | **HR^†^** | **95% CI** | ***p*** |
| **Risk factors** |  |  |  |  |  |  |  |  |  |  |  |
| Age (per years) | 1.04 | 1.03 - 1.06 | < 0.001 |  | 1.03 | 1.01 - 1.05 | <0.001 |  | 1.03 | 1.01 - 1.05 | < 0.001 |
| Male sex | 0.77 | 0.58 - 1.03 | 0.076 |  | 0.93 | 0.66 - 1.31 | 0.660 |  | 0.90 | 0.64 - 1.28 | 0.562 |
| Hypertension | 1.28 | 0.96 - 1.72 | 0.091 |  | 0.88 | 0.64 - 1.20 | 0.414 |  | 0.91 | 0.66 - 1.25 | 0.554 |
| Diabetes mellitus | 1.77 | 1.35 - 2.33 | < 0.001 |  | 1.51 | 1.13 - 2.03 | 0.006 |  | 1.59 | 1.19 - 2.13 | 0.002 |
| Coronary artery disease | 1.57 | 1.14 - 2.15 | 0.005 |  | 1.31 | 0.93 - 1.83 | 0.121 |  | 1.30 | 0.93 - 1.81 | 0.127 |
| Atrial fibrillation | 2.06 | 1.30 - 3.27 | 0.002 |  | 1.68 | 1.04 - 2.71 | 0.033 |  | 1.73 | 1.07 – 2.80 | 0.026 |
| Chronic kidney disease | 2.31 | 1.49 - 3.60 | < 0.001 |  | 1.44 | 0.80 - 2.59 | 0.222 |  | 1.55 | 0.87 - 2.75 | 0.136 |
| Previous stroke | 1.96 | 1.31 - 2.94 | 0.001 |  | 1.61 | 1.03 - 2.53 | 0.036 |  | 1.61 | 1.03 - 2.52 | 0.036 |
| Current smoker | 0.73 | 0.54 -1.00 | 0.050 |  | 1.05 | 0.74 - 1.48 | 0.782 |  | 1.09 | 0.77 - 1.54 | 0.632 |
| Hemoglobin | 0.89 | 0.85 - 0.94 | < 0.001 |  | 0.96 | 0.90 - 1.03 | 0.293 |  | 0.96 | 0.90 - 1.03 | 0.254 |
| **Inter-arm and -leg BP difference** | | | | | | | | | |  |  |
| Arm SBP Difference, per 5 mmHg | 1.09 | 0.93 - 1.28 | 0.275 |  | 1.08 | 0.92 – 1.27 | 0.350 |  |  |  |  |
| Arm DBP Difference, per 5 mmHg | 0.95 | 0.70 - 1.29 | 0.746 |  |  |  |  |  | 0.88 | 0.65 – 1.20 | 0.428 |
| Leg SBP Difference, per 5 mmHg | 1.13 | 1.06 - 1.19 | < 0.001 |  | 1.07 | 1.00 - 1.14 | 0.039 |  |  |  |  |
| Leg DBP Difference, per 5 mmHg | 1.10 | 1.00 - 1.20 | 0.048 |  |  |  |  |  | 1.01 | 0.91 - 1.13 | 0.838 |

BP, blood pressure; CI, confidence interval; DBP, diastolic blood pressure; HR, hazard ratio; SBP, systolic blood pressure.

**^*^**Adjusted with risk factors, arm SBP difference, and leg SBP difference.

**^†^**Adjusted with risk factors, arm DBP difference, and leg DBP difference.
