# Supplementary material for: Association between inter-leg blood pressure difference and cardiovascular outcome in patients undergoing percutaneous coronary intervention
Source: PLoS One. 2021 Oct 15;16(10):e0257443. doi: 10.1371/journal.pone.0257443 (PMC8519463; doi:10.1371/journal.pone.0257443)
Supplement: S3 Table — (DOCX) [file pone.0257443.s005.docx]

**S3 Table. Comparison of characteristics between patients with ILSBPD <16mmHg and ILSBPD ≥ 16mmHg**

| **Variables** | **ILSBPD <16 mmHg** | **ILSBPD ≥16 mmHg** | ***p* value** |
| --- | --- | --- | --- |
|  | **(n = 595)** | **(n = 72)** |  |
| Age (years) | 63.5 ± 10.9 | 68.3 ± 11.5 | 0.001 |
| Male sex | 418 (70.3) | 52 (72.2) | 0.834 |
| Body mass index | 24.8 ± 3.6 | 23.4 ± 3.1 | 0.001 |
| Hypertension | 361 (60.7) | 55 (76.4) | 0.013 |
| Diabetes mellitus | 173 (29.1) | 32 (44.4) | 0.011 |
| Coronary artery disease | 105 (17.6) | 17 (23.6) | 0.282 |
| Atrial fibrillation | 37 (6.2) | 64 (8.3) | 0.663 |
| Chronic kidney disease | 24 (4.0) | 12(16.7) | <0.001 |
| Previous stroke | 43 (7.2) | 9 (12.5) | 0.179 |
| Current smoking | 182 (30.6) | 20 (27.8) | 0.716 |
| Hemoglobin (g/dL) | 13.4 ± 2.3 | 12.6 ± 2.4 | 0.006 |
| LDL (mg/dL) | 100.9 ± 37.9 | 89.3 ± 32.0 | 0.016 |
| HDL (mg/dL) | 42.3 ± 12.3 | 39.8 ± 9.5 | 0.052 |
| LVEF (%) | 61.6 ± 11.0 | 60.7 ± 12.1 | 0.525 |

Data showed by mean (± SD), or number (%). HDL, high-density lipoprotein; ILSBPD, inter-leg systolic blood pressure difference; ILDBPD, inter-leg diastolic blood pressure difference; LDL, low-density lipoprotein; LVEF, left ventricular ejection fraction.
